# A Standardized Method for 4D Ultrasound-Guided Peripheral Nerve Blockade and Catheter Placement

**DOI:** 10.1155/2014/920538

**Published:** 2014-01-19

**Authors:** N. J. Clendenen, C. B. Robards, S. R. Clendenen

**Affiliations:** ^1^Department of Anesthesiology, Yale University School of Medicine, 333 Cedar Street, New Haven, CT 06520, USA; ^2^Department of Anesthesiology, Mayo Clinic, 4500 San Pablo Boulevard, Jacksonville, FL 32224, USA

## Abstract

We present a standardized method for using four-dimensional ultrasound (4D US) guidance for peripheral nerve blocks. 4D US allows for needle tracking in multiple planes simultaneously and accurate measurement of the local anesthetic volume surrounding the nerve following injection. Additionally, the morphology and proximity of local anesthetic spread around the target nerve is clearly seen with the described technique. This method provides additional spatial information in real time compared to standard two-dimensional ultrasound.

## 1. Introduction

Conventional two-dimensional (2D) ultrasound guidance during peripheral catheter placement is quickly becoming common practice due to advantages over blind or nerve stimulator guided techniques, namely, needle tracking and visualization of local anesthetic spread. However, substantial challenges in ultrasound guided regional anesthesia remain such as maintaining the needle tip in view during needle advancement and confirming catheter placement.

Real-time three-dimensional (4D) ultrasound offers several potential advantages compared to 2D imaging for guiding peripheral nerve block placement. First, 4D ultrasound imaging allows for simultaneous visualization of multiple planes of view, thereby permitting longitudinal, cross-sectional, and coronal images without probe adjustment. Second, it provides additional information about the spatial relationship between anatomical structures of interest compared to standard imaging, which could aid in preventing needle misadventure. Additionally, 4D ultrasound also allows for accurate volume measurements which are useful for measuring local anesthetic spread and tracking fluid dissipation in a fascial compartment over time. Recently, a method for static localization of needle and catheter placement for regional anesthesia has been described with postacquisition volume rendering of 3D ultrasound [1] which provides additional information but does not permit 3D needle tracking in real time. Radiologists have employed 4D ultrasound guided nerve tracking and target localization for solid tumor biopsies with success [2, 3]. Also, case reports have demonstrated the feasibility of 4D ultrasound guided peripheral nerve blockade [4, 5] and thoracic paravertebral anesthesia [6]; however, a standard approach using this new technology has not been described. In this report we present a standardized method for 4D guided peripheral nerve blocks and catheter placement.

## 2. Materials

 Philips iU22 Ultrasound Imaging System
 Philips X6-1 xMatrix Array Transducer
 Philips Qlab Quantification Software.

## 3. Methods

1. Orient the probe in a transverse position to the path of the nerve. Increase the field of view by widening the sector width. Optimize for the best cross-sectional view of the nerve or vessel by adjusting the focus and gain and using XRES and harmonics.
2. Activate x-Plane and “right invert.” The view established in step 1 will appear in the primary window on the left. Using lateral tilt adjust the reference line in the primary window with the track ball so that it intersects the nerve. A longitudinal view of the nerve will be visible in the secondary plane on the right.
3. Insert the needle in line with the center of the probe at approximately a 45- to 60-degree angle. Identify the needle in the primary plane (short axis view) and again use the lateral tilt to bring the needle into view in the secondary plane (long axis view).
4. Advance the needle to the target while keeping the needle in view.
5. Start 4D and select the 4 panel view showing transverse, longitudinal, coronal, and volume view. With the MPR crosshair set to partial, adjust the crosshair so that it lies over the tip of the needle in the transverse, longitudinal, and coronal view.
6. Draw back on the syringe to ensure that the needle is not intravascular and inject a test dose of local anesthetic. Confirm that the local anesthetic spreads appropriately by enveloping the nerve. Inject the total volume if a single dose nerve block is desired or 2/3 of the total local anesthetic dose if inserting a catheter. The spread of the local anesthetic in all four planes (transverse, longitudinal, coronal, and 3D volume) will be seen and the nerve should now be clearly visible due to the fluid-nerve interface.
7. Thread the catheter 2 to 5 cm past the needle tip.
8. Locate the catheter tip by agitating the final 1/3 of local anesthetic and injecting it through the catheter. The agitated local anesthetic will emerge from the catheter tip and appear as a hyper-echoic cloud.
9. Once the total volume of local anesthetic is given, acquire a data set by pressing “save 3D volume”.
10. Launch Q-lab to measure the approximate volume of local anesthetic in the perineural space.
11. In Q-lab, adjust the threshold to better visualize the fluid/tissue interface. Press “calliper” on the touch screen and outline the local anesthetic spread with the ellipse tool. Use the track ball to adjust the calliper and measure the diameter of the fluid space. The volume calculation appears on the screen.

The patients undergoing a nerve block demonstrated in this work provided informed consent and received standard pre- and postoperative care for elective orthopaedic procedures unrelated to the study. The images are anonymized to protect their privacy.

## 4. Results

**Figure 1:**
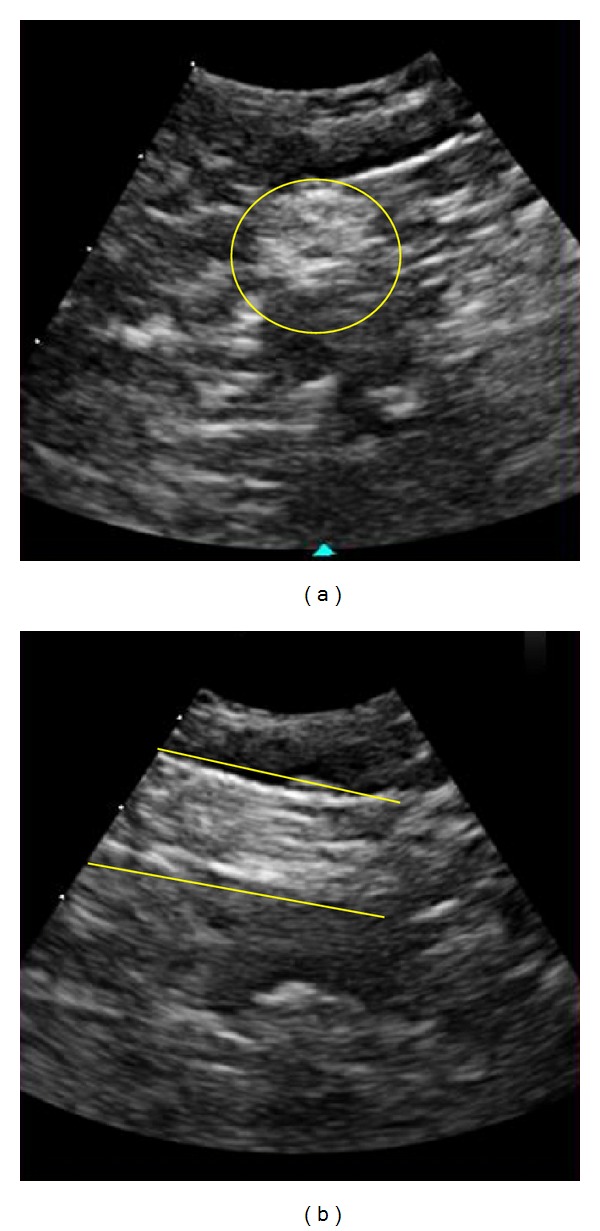
Image of the sciatic nerve at the popliteal fossa in short and long axis view obtained simultaneously in x-Plane mode. The yellow outlines are added for clarity.

**Figure 2:**
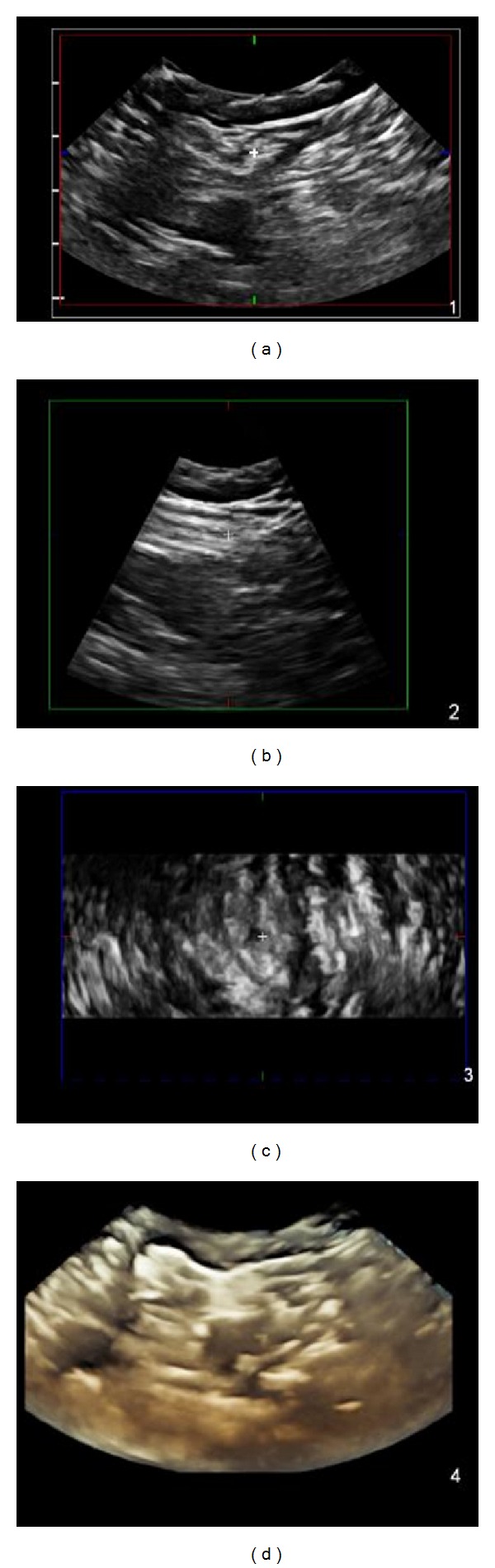
Standard view of the sciatic nerve from the popliteal fossa in 4D mode. The white crosshair corresponds to the same location on the sciatic nerve in short axis (a), long axis (b), and coronal view (c). (d) is a three-dimensional composite image that updates in real time.

**Figure 3:**
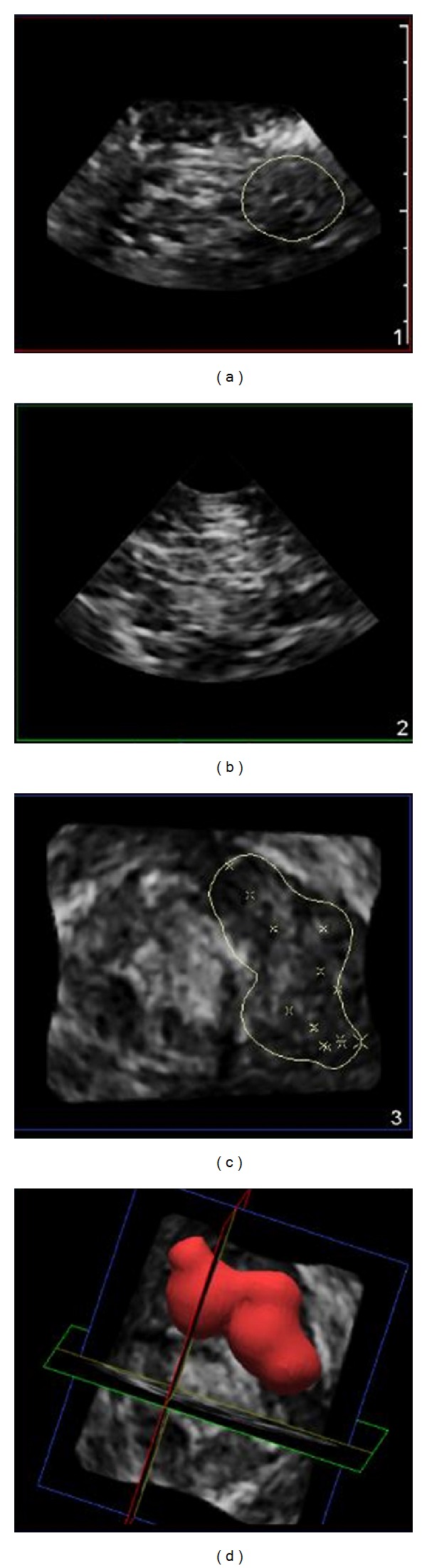
Infraclavicular brachial plexus block volume calculated as 21.2 milliliters demonstrating epineural local anesthetic spread. Injected local anesthetic volume was 20 milliliters.

**Figure 4:**
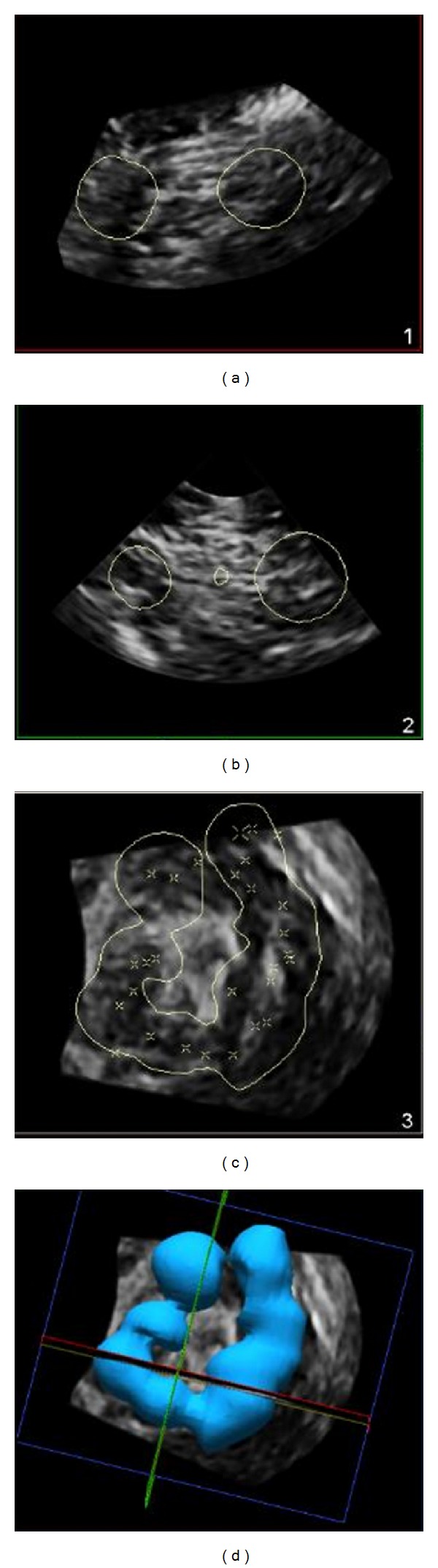
Infraclavicular brachial plexus block volume calculated as 41 milliliters demonstrating circumneural local anesthetic spread. Injected local anesthetic volume was 40 milliliters.

Orienting the ultrasound probe perpendicular to the nerve provides a clear view of relevant anatomical landmarks and allows for planning the needle's path to the perineural space. Figure 1 shows the standardized view of the sciatic nerve from the popliteal fossa in short and long axis in biplane mode. Inserting the needle at approximately a 45- to 60-degree angle increases the likelihood of viewing the needle in the initial 2D cross-sectional scan. Advancing the needle in the biplane view allows for simultaneous in plane and out of plane imaging of the needle approach. The needle is first imaged as a hyperechoic point with acoustic shadowing that moves in response to needle manipulation. In x-Plane mode, adjusting the tilt of the probe so that the line intersects the cross-section of the needle allows a longitudinal view of the needle to be clearly seen in the second x-Plane window. After switching to the 4D mode with four windows, the MPR crosshair must be adjusted to keep the needle in view in each plane and confirm appropriate positioning prior to injection.  Figure 2 is an example of the sciatic nerve from the popliteal fossa in 4D mode. Injected local anesthetic will appear hypoechoic as it opens the perineural potential space. The catheter appears as a thin moderately echogenic structure against the background of hypoechoic local anesthetic in the 3D volume view, which allows for tracing its course in the perineural space. Viewing agitated local anesthetic as it emerges from the catheter tip provides a means of confirming the location of the catheter and ensures that it will deliver local anesthetic to the appropriate space for postoperative analgesia. The agitated local anesthetic will appear as a hyper-echoic cloud in the perineural space. With a 3D volume data set, the amount of local anesthetic present in the perineural space following injection may be measured by using the “calliper” function after 3D image acquisition. The 3D volume data set allows for differentiation between an epineural and circumneural local anesthetic spread and volume measurements within 10% of the known injection volume (Figures 3 and 4).

## 5. Discussion

Real-time three-dimensional ultrasound imaging (4D US) is a useful tool for guiding placement of peripheral nerve blocks for postoperative analgesia. The standardized method described in this report offers a reproducible and effective approach to 4D US guided regional anesthesia. Using advanced ultrasound guided techniques may address current challenges of regional anesthesia such as needle tracking, locating the catheter tip, and measuring local anesthetic spread in a fascial plane. Localizing the catheter tip by imaging the injection of agitated local anesthetic was reported previously with two-dimensional ultrasound imaging [7] and we have adapted the technique for 4D imaging. Additionally, echogenic needles are an emerging tool for clearly visualizing a needle and its tip during peripheral nerve block placement and have been shown to decrease the time required for block placement in one study [8]. Electromagnetic needle tracking is another novel system for visualizing and predicting the needle path during peripheral nerve blockade [9]. Combining these technical developments with 4D ultrasound may lead to a synergistic improvement in imaging during peripheral nerve blockade. Current limitations to widespread use of 4D US are that a specialized machine and transducer are required. Additionally, 4D capable transducers are lower frequency than the best performing 2D transducers due to the technical challenges of arranging the dense array of over 9,000 piezoelectric elements onto the transducer head that allows for real-time 3D imaging [10]. Clearly identifying the spatial relationship between nerves and vasculature with 4D US may also allow clinicians to avoid complications such as intravascular injection of local anesthetics. A recent prospective study demonstrated the utility of 3D ultrasound imaging of local anesthetic spread to identify needle placement and subfascial versus extrafascial injection during placement of a lateral popliteal nerve block [11]. Further well-designed clinical trials are necessary to determine whether the additional imaging capabilities translate into a reduction of adverse events and improved clinical outcomes while clarifying the differences between techniques in regional anesthesia. An earlier case report highlights the advantages of a multiplanar approach during a single shot radial nerve block [12]. We sought to formalize this approach into a standardized method for 4D ultrasound guidance of needle placement in addition to describing a method for viewing and calculating injected local anesthetic volume. Future studies employing this method may enable additional qualitative and quantitative assessment of techniques in regional anesthesia.

## Supplementary Material

The supplementary material is a video demonstrating the standardized method for 4D ultrasound guided nerve blockade described in the manuscript. Note the multiple views that are simultaneously displayed during needle advancement and local anesthetic injection. The video is presented at the acquisition frame rate and punctuated by labelled still images for clarity.
